# Deletion of the Innate Immune NLRP3 Receptor Abolishes Cardiac Ischemic Preconditioning and Is Associated with Decreased Il-6/STAT3 Signaling

**DOI:** 10.1371/journal.pone.0040643

**Published:** 2012-07-27

**Authors:** Coert J. Zuurbier, Willeke M. C. Jong, Otto Eerbeek, Anneke Koeman, Wilco P. Pulskens, Loes M. Butter, Jaklien C. Leemans, Markus W. Hollmann

**Affiliations:** 1 Laboratory of Experimental Intensive Care and Anesthesiology, Department of Anesthesiology, Academic Medical Center, University of Amsterdam, Amsterdam, The Netherlands; 2 Department of Physiology, Academic Medical Center, University of Amsterdam, Amsterdam, The Netherlands; 3 Department of Pathology, Academic Medical Center, University of Amsterdam, Amsterdam, The Netherlands; McGill University, Canada

## Abstract

**Objective:**

Recent studies indicate that the innate immune system is not only triggered by exogenous pathogens and pollutants, but also by endogenous danger signals released during ischemia and necrosis. As triggers for the innate immune NLRP3 inflammasome protein complex appear to overlap with those for cardiac ischemia-reperfusion (I/R) and ischemic preconditioning (IPC), we explored the possibility that the NLRP3 inflammasome is involved in IPC and acute I/R injury of the heart.

**Principal Findings:**

Baseline cardiac performance and acute I/R injury were investigated in isolated, Langendorff-perfused hearts from wild-type (WT), ASC^−/−^ and NLRP3^−/−^ mice. Deletion of NLRP3 inflammasome components ASC^−/−^ or NLRP3^−/−^ did not affect baseline performance. The deletions exacerbated I/R-induced mechanical dysfunction, but were without effect on I/R-induced cell death. When subjected to IPC, WT and ASC^−/−^ hearts were protected against I/R injury (improved function and less cell death). However, IPC did not protect NLRP3^−/−^ hearts against I/R injury. NLRP3^−/−^ hearts had significantly decreased cardiac IL-6 levels with a trend towards lower IL-1β levels at end reperfusion, suggesting abrogation of IPC through diminished IL-6 and/or IL-1β signaling. Subsequent experiments showed that neutralising IL-6 using an antibody against IL-6 abrogated IPC in WT hearts. However, inhibition of the IL-1r receptor with the IL-1 receptor inhibitor Anakinra (100 mg/L) did not abrogate IPC in WT hearts. Analysis of survival kinases after IPC demonstrated decreased STAT3 expression in NLRP3^−/−^ hearts when compared to WT hearts.

**Conclusions:**

The data suggest that the innate immune NLRP3 protein, in an NLRP3-inflammasome-independent fashion, is an integral component of IPC in the isolated heart, possibly through an IL-6/STAT3 dependent mechanism.

## Introduction

The innate immune system is the first line of defence against stress signals such as exogenous pathogen-associated molecular patterns (PAMPs) and pollutants. Intriguingly, recent data have demonstrated that the innate immune system is also activated by ischemia and necrosis through endogenous danger-associated molecular patterns (DAMPs), the so-called sterile inflammatory response [1]. Such DAMPs may entail uric acid, adenosine, ATP, heat shock protein, HMGB1, DNA, or myosin released by damaged cells [1]–[3]. Interestingly, it is suggested that these DAMPs activate the cellular innate immune system through trigger mechanisms involving potassium extrusion and radical production [4], [5]. Exact similar trigger mechanisms are implicated in cardiac ischemia-reperfusion (I/R) and ischemic preconditioning (IPC) [6], suggesting that activation of the innate immune system is maybe an intrinsic part of I/R and IPC physiology. In the current work we examine to what extent the NLRP3 inflammasome, a specific part of the innate immune system, affects acute I/R and IPC cardiac physiology. Such interrelationships between hypoxia, IPC and inflammation are also well documented for other, non-inflammasome, parts of the immune system, where hypoxia-induced hypoxia-inducible transcription factor HIF modulates inflammation and IPC through adenosine and NF-κB signaling [7], [8].

Innate immune responses are activated within minutes upon encounter with DAMPs or PAMPs. Such receptors entail the well-known Toll-like receptors (TLR), localized either at the cell surface or within endosomes, and the nucleotide oligomerization domain (Nod)-like receptors (NLRs), which are intracellular cytosolic sensors [9]. NLRP3 inflammasome, a member of the NLRs, is a multiprotein complex consisting of NLRP3, along with ASC (adapter apoptosis-associated speck-like protein containing a C-terminal CARD) and caspase-1 [10]. Upon assembly, caspase-1 is activated resulting in the processing and release of proinflammatory cytokines among which the interleukin IL-1β figures prominently [9].

The NLRP3 inflammasome is critically involved in the sterile inflammatory response as reported for e.g. monocytes and tumour cells [4], [10], [11]. In addition, it has recently been shown that NLRP3 deficiency protects animals against renal ischemic tubular necrosis [12]. Inflammation is also critically involved in myocardial I/R injury, with a prominent role for IL-1β as an early mediator of inflammation [13], [14]. Our first goal is therefore to examine the role of the NLRP3 inflammasome in acute myocardial I/R injury, knowledge that is currently missing in the literature. Conversely, IL-1β can indirectly modulate IL-6 and TNF-alpha [15]. These inflammatory mediators may also be protective, because they are able to induce IPC [16], [17]. Moreover, mitochondrial signals [5], [6], [12] seem to mediate both activation of the NLPR3 inflammasome and IPC protective effects in relation to I/R injury. It therefore seems possible that NLRP3 inflammasome activation may interact with IPC. To our knowledge, no information is available whether the NLRP3 inflammasome is involved in IPC. Our second goal is therefore to examine the role of the NLRP3 inflammasome in cardiac IPC. Finally, although initial research has emphasized the importance of the formation of the multiprotein complex NLRP3 inflammasome for their inflammatory effects, suggesting that the individual components only exert its action through complex formation, very recent research has indicated complex ( = inflammasome)-independent effects of the separate components [18], [19]. Because such information is currently lacking for cardiac I/R and IPC, we also addressed this question through comparison of NLRP3 gene and ASC gene knockout mice.

In this study we have investigated whether the NLRP3 inflammasome is involved in acute cardiac I/R injury and IPC, and whether specific inflammatory pathways that are affected by ablation of constituents (NLRP3 or ASC) of the assembled NLRP3 inflammasome are involved in observed changes in I/R and IPC.

## Methods

### Animals

C57BL/6J Nlrp3^−/−^ and ASC^−/−^ mice were originally obtained from dr RA Flavell (Yale University, New Haven, CT, USA), and described before [20]. Both genotypes were backcrossed with C57BL/6J background for at least 9 generations. The wild-type (WT) mice in the present study were all C57BL/6J obtained from Charles River. Experiments were performed with male mice only. Mice were fed a standard chow (CRM(E) diet, SDS, Witham, England) *ad libitum* and studied at 10–15 weeks of age. All experiments were approved by the animal ethics committee of the Academic Medical Center, Amsterdam, The Netherlands.

### Heart Perfusion

In vitro I/R protocol was performed as previously described with slight modifications [21], [22]. Mice were heparinized (15 IU) and anesthetized with Nembutal (80 mg kg^−1^). Following tracheotomy, the mice were mechanically ventilated and a thoracotomy performed. The hearts were cannulated in situ with perfusion started before excision of the heart. Hearts were Langendorff-perfused at a constant flow (initial perfusion pressure 80 mm Hg) at 37°C with Krebs-Henseleit solution containing (mmol l^−1^) NaCl 118, KCl 4.7, CaCl_2_ 2.25, MgSO_4_ 1.2, NaHCO_3_ 25, KH_2_PO_4_ 1.2, EDTA 0.5 and glucose 11, gassed with 95% O_2_/5% CO_2_. The perfusate was in-line filtered by a 0.45-µm filter. End-diastolic pressure (EDP) was set at ∼4–8 mmHg using a water-filled polyethylene balloon inserted into the left ventricular (LV) cavity via the mitral valve. The hearts were continuously submerged in 37°C perfusate. LV developed pressure was calculated as the systolic pressure (Psys) minus EDP. The rate-pressure product (RPP, index of mechanical performance)) was the product of the developed LV pressure and the heart rate. Following 20 min stabilization of LV pressure, all hearts were subjected to 35 min of perfusion with or without IPC, followed by 35 min no-flow ischemia and 45 min reperfusion. During ischemia, the hearts were submerged in Krebs-Henseleit perfusate gassed with 95% N_2_/5% CO_2_. The IPC protocol consisted of 3 times 5 min ischemia followed by 5 min reperfusion for the first two times of 5 min I, and 10 min reperfusion following the last 5 min I, before the 35 min of I.

In the first series of experiments, six different groups of hearts (n = 7 each) were studied consisting of WT, ASC^−/−^ and NLRP3^−/−^ genotypes, with each genotype subjected to either I/R only or to I/R plus IPC.

In the second series of experiments, two groups were examined (n = 7 each) to explore potential NLRP3 cytokine-related pathways in IPC. In the first group, WT hearts were continuously exposed from the start of the experiment to 100 mg/L Anakinra (Kineret, Biovitrum; an interleukin 1 receptor (IL-1 RA) inhibitor during IPC, to examine a possible role for IL-1 signaling in acute IPC. In the second group, WT hearts were continuously exposed from the start of the experiment to 50 ng/ml anti-mouse IL-6 antibody (R&D systems), to neutralize a possible role for IL-6 release and signalling in IPC.

At the end of these experiments, hearts were weighed and immediately homogenized in 1.5 ml homogenization medium (in mM: 250 sucrose, 20 Hepes (pH 7.4), 10 KCl, 1.5 MgCl_2_, 1 EDTA, 0.1 PMSF, 5 µg/ml leupeptin, 5 µg/ml aprotinin and 1 µg/ml pepstatin). Homogenates were frozen at −80°C until further analysis for cytokines and mRNA expression levels.

Finally, in a third series of experiments, two additional groups (n = 4 each) were studied, to examine whether IPC in NLRP3^−/−^ hearts was associated with alterations in well-known cardioprotective survival pathways. To this end, isolated hearts from WT and NLRP3^−/−^ animals were subjected to IPC and global ischemia as described above. At 5 min reperfusion hearts were immediately freeze-clamped and stored at −80°C until further analysis of survival kinases.

**Table 1:** Physiological characteristics of Langendorff-perfused hearts of wild-type (WT), ASC^−/−^ and NLRP3^−/−^ mice.

	Flow (ml/min/g)	EDP (mm Hg)	Psys (mm Hg)	HR (beats/min)	RPP (10^3^ mmHg/min)
WT	14.4±0.7	6.1±0.5	118±2	376±11	42.0±1.6
ASC^−/−^	14.3±1.4	5.7±0.4	119±4	370±14	42.3±2.4
NLRP3^−/−^	13.8±1.6	6.4±0.6	117±3	359±9	39.7±1.5

Values are given as (mean ± SEM). EDP: end-diastolic pressure; Psys: peak systolic pressure; HR: heart rate; RPP: rate-pressure-product.

### Lactate Dehydrogenase Enzyme Activity in Effluent

During the reperfusion period the effluent was collected at 5, 10, 15, 30 and 45 min of reperfusion and immediately frozen at −80°C. Lactate dehydrogenase (LDH) activity was determined using standard spectrophotometric analysis at 340 nm [23]. LDH release is used as index of necrosis, as other studies have shown a good correlation between LDH release and TTC staining [24], [25].

### Cytokine Determination in Hearts

Homogenate was treated with 0.5% Triton X-100 for 10 min, centrifuged at 10,000 g for 1 min and supernatant was used for further analysis. Cytokines were determined in the supernatant by ELISA: TNF-α (Quantikine Mouse TNF-α, R&D systems), IL-1β (Quintikine Mouse IL-1β, R&D systems) and IL-6 (Duoset Mouse IL-6, R&D systems). Results are normalized to protein content determined by the Bradford method.

**Figure 1:**
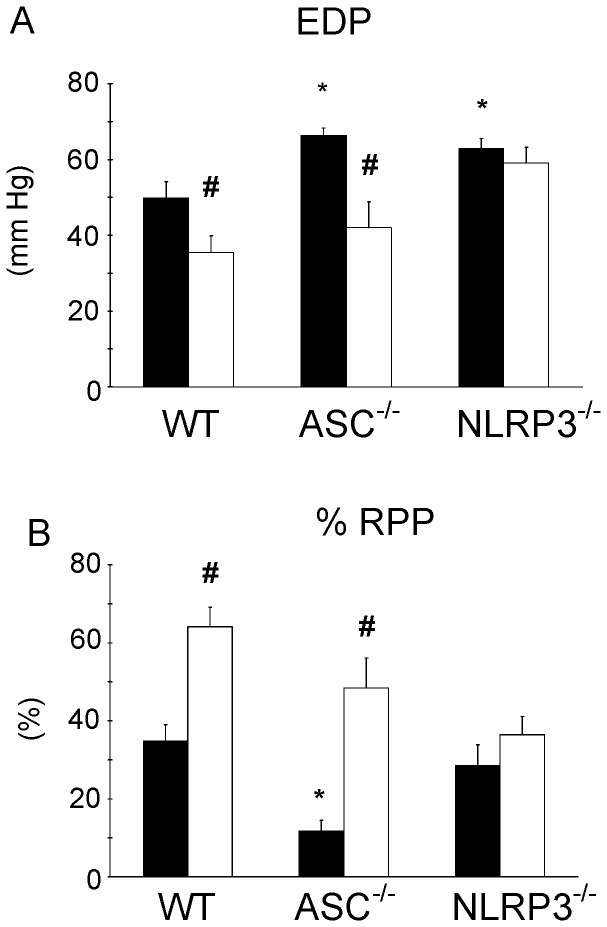
Ablation of NLRP3, but not ASC, abrogated IPC protective effects on cardiac function following I/R. Effect of ASC and NLRP3 gene ablation on cardiac mechanical performance following acute I/R with or without preceding IPC in isolated perfused mouse hearts. (A) End-diastolic pressure (EDP) measured at end reperfusion for the different groups; (B) % Rate-pressure product (RPP) determined at end reperfusion and normalized to baseline, pre-ischemic, values for the different groups. Black bars denote I/R groups, white bars reflect IPC + I/R groups. (n = 7 for all groups). Mean ± SEM, * P<0.05 vs. I/R in WT, ^#^ P<0.05 IPC+I/R vs. I/R similar group (ANOVA).

### HMGB1 mRNA Expression Analysis in Hearts

Homogenate was treated with 0.5% Triton X-100 for 10 min, centrifuged at 10,000 g for 1 min and supernatant was used for further analysis. Total RNA was extracted from heart tissue with Trizol reagent (Invitrogen) according to the manufacturer’s protocol. All RNA samples were quantified by spectrophotometry and stored at −80°C until processed for reverse transcription. RNA was converted to cDNA by using oligo-dT as primer. High mobility group box (HMGB)1 mRNA expression was analyzed by RT-PCR performed on a Roche light cycler with SYBR green PCR master mix. Specific gene expression was normalized to mouse peptidylpropyl isomerase A (PPIA) gene expression. SYBR green dye intensity was analyzed with linear regression analysis. Primer sequences (Biolegio) were as follows: PPIA forward primer, 5′-tgccagggtggtgactttac; reverse primer, 5′-gatgccaggacctgtatgct; HMGB1 forward primer, 5′-gagagatgtggaacaacactgc; reverse primer, 5′-ctgtaggcagcaatatccttctc.

**Figure 2:**
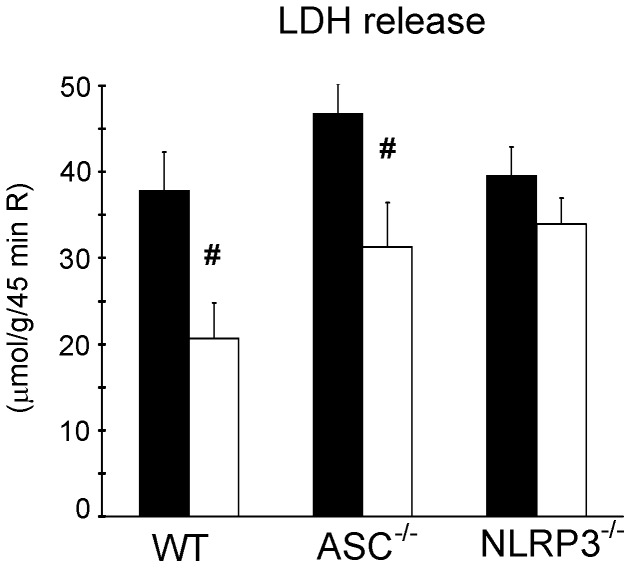
Ablation of NLRP3, but not ASC, abrogated IPC protective effects on cardiac I/R-induced cell death. Effects of ASC and NLRP3 gene ablation on cell death (LDH enzyme release) after I/R with or without preceding IPC. Accumulative LDH release during 45 min reperfusion is given for the different groups. Black bars denote I/R groups, white bars reflect IPC + I/R groups. (n = 7 for all groups). Mean ± SEM, * P<0.05 vs. I/R in WT, ^#^ P<0.05 IPC+I/R vs. I/R similar group (ANOVA).

### Survival Kinases in Preconditioned, Reperfused Hearts

Total heart homogenate was obtained by grinding a snap-frozen heart in liquid nitrogen into powder, followed by homogenization on ice in 1.5 ml homogenate buffer (in mM: 20 Hepes (pH 7.4), 70 NaCl, 2.5 MgCl_2_, 1 EDTA, 0.1 PMSF, 0.5% Triton, 0.1 PMSF, 5 µg/ml leupeptin, 5 µg/ml aprotinin, 1 µg/ml pepstatin and phosphatase inhibitors. The homogenate was kept on ice for 60 min with intermittent vortex mixing. Finally, the mixture was homogenized again on ice, and stored at −80°C. Equal amounts of homogenate protein were electrophoresed on a precast gels (4–12% Bis-Tris, Criterion XT, Bio-Rad), and transferred to an Immobilon-FL Membrane (Type PVDF, pore size 0.45µm, Millipore). The membranes were blocked with ready to use blocking buffer (#927-40000, Li-Cor) for 1 hour. To detect phosphorylated proteins, the membranes were incubated at 4°C overnight with antibodies against pSTAT3 (Tyr 705) (1∶500; Cell Signaling), pAMPKα (1∶2500; Cell Signaling), p-p44/42 MAP kinase (1∶5000; Cell Signaling) and pPKCε (1∶2500; Bio connect). To detect total proteins, the membranes were incubated at 4°C overnight with antibodies against STAT3 (1∶1000; Cell Signaling), AMPKα (1∶5000; Cell Signaling), p44/42 MAP kinase (1∶10000; Cell Signaling), against PKCε (1∶5000; Bio connect), and against α-tubulin (1∶20000; Sigma). After washing 3× with PBS with 0.1% Tween 20, membranes were incubated for 1 hour with secondary antibodies labelled with IRDye infrared dyes. Following washing, membranes were analyzed with the Odyssey Infrared Imaging System (Li-Cor). Densities of survival proteins were normalized to the density of α-tubulin.

#### Statistical analysis

Data are presented as means ± SE. Data were analyzed ANOVA followed by Fisher’s post hoc tests. Values of P<0.05 were considered to be statistically significant.

**Figure 3:**
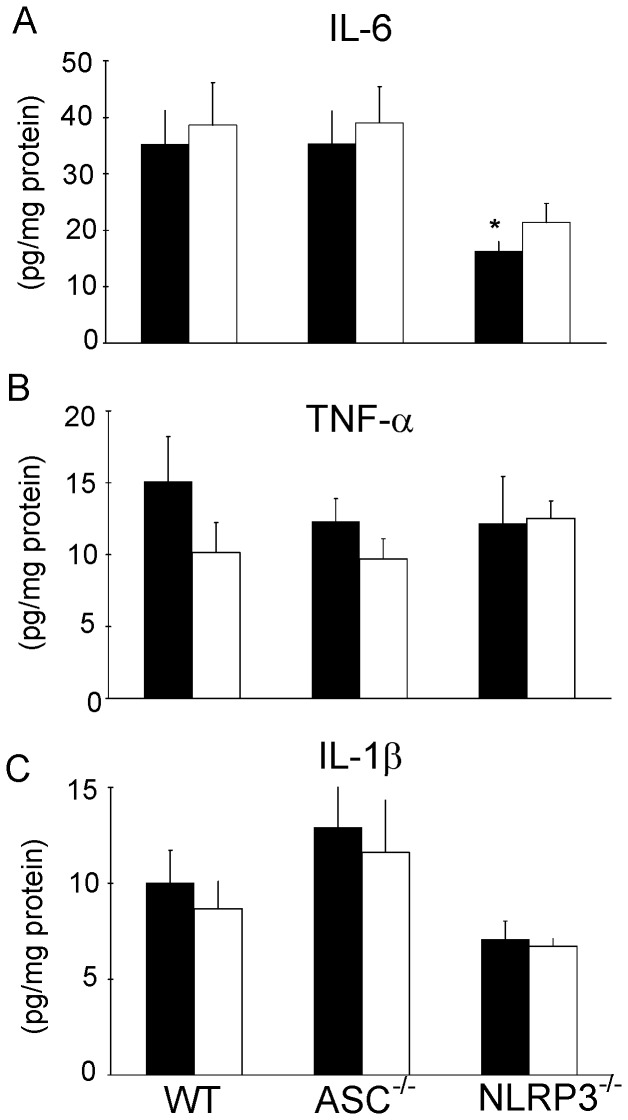
Reduced IL-6 cytokine levels in reperfused NLRP3^−/−^ hearts. Myocardial cytokine levels at end reperfusion for wild-type (WT), ASC^−/−^ and NLRP3^−/−^ hearts subjected to I/R with or without IPC. IL-6 (A), TNF-α (B) and IL-1β (C) are given. Black bars denote I/R groups, white bars reflect IPC + I/R groups. (n = 7 for all groups). Mean ± SEM, * P<0.05 vs. I/R in WT (ANOVA).

## Results

Genetic manipulations of the NLRP3 inflammasome components were without effect on baseline cardiac physiological parameters of the isolated heart (table 1). No differences in the RPP or flow were found in the conglomerate of HR and peak developed LV pressure, indicating that basal work performed by the hearts was not affected by deletion of the NLRP3 or ASC component of the NLRP3 inflammasome.

**Figure 4:**
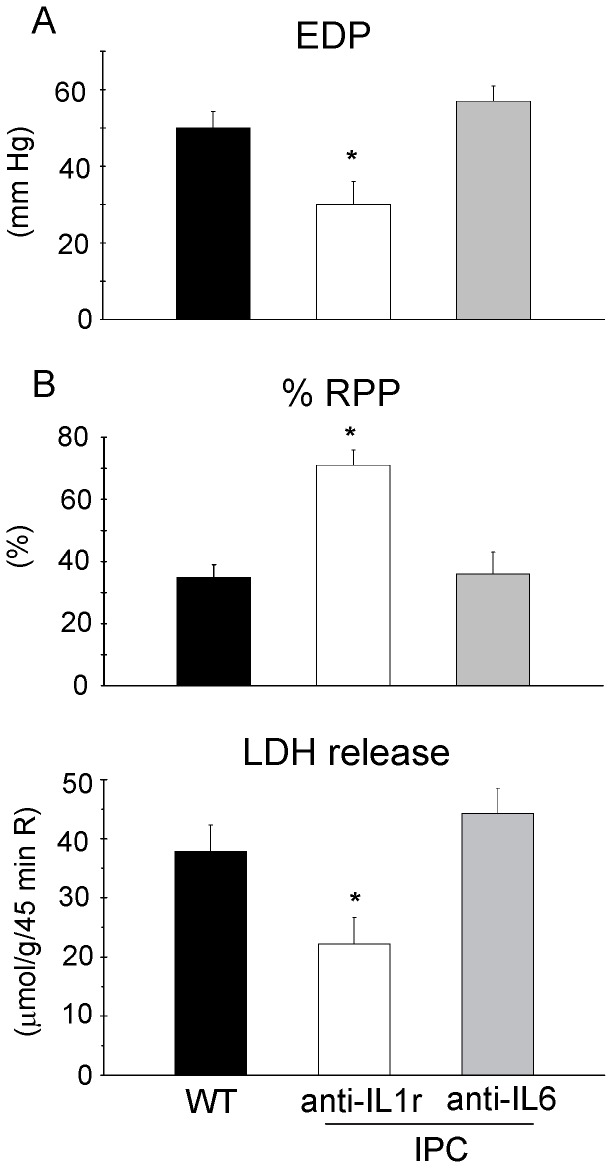
Neutralizing IL-6, but not inhibition of IL-1r receptor, prevented IPC protection in wild-type hearts. Effect of inhibition of IL-1R receptor and neutralization of IL-6 on cardioprotective effect of IPC in WT hearts. IPC protective effects were evaluated for EDP (A), RPP (B) and LDH release during reperfusion (C). (n = 7 for all groups). Mean ± SEM, * P<0.05 WT+I/R+IPC+inhibition vs. WT+I/R (ANOVA).

To evaluate I/R-induced lethal injury, we examined three classical parameters for lethal injury, i.e. contracture ( =  EDP of the heart), mechanical performance ( =  RPP) and cell death (release of the enzym lactate dehydrogenase in the effluent). The I/R intervention resulted in 50±4 mm Hg EDP (Fig. 1A) and 35±4 % recovery of the RPP (Fig. 1B) in WT hearts at the end of 45 min reperfusion. To examine whether NLRP3 inflammasome components ASC and NLRP3 affected I/R-induced mechanical dysfunction, ASC^−/−^ and NLRP3^−/−^ hearts were also subjected to I/R and compared to WT hearts. ASC^−/−^ hearts showed increased I/R injury on these functional cardiac parameters as compared to WT hearts: EDP was increased to 66±2 mm Hg, and RPP recovery decreased to 12±3%. The NLRP3^−/−^ hearts only demonstrated increased I/R-induced contracture (63±3 mmHg) as compared to WT hearts, without affecting RPP recovery. We conclude that both the ASC and NLRP3 component of the NLRP3 inflammasome complex contribute to improved heart function immediately following an I/R insult.

**Figure 5:**
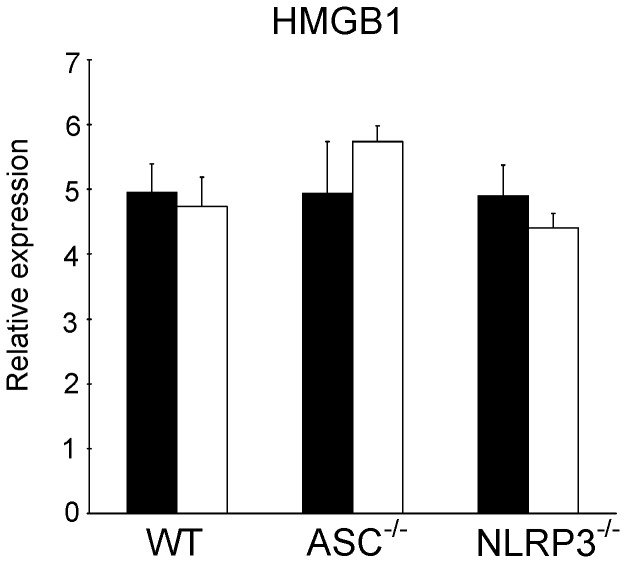
Unaltered HMGB1 mRNA levels. Myocardial HMGB1 mRNA levels at end reperfusion for wild-type (WT), ASC^−/−^ and NLRP3^−/−^ hearts subjected to ischemia-reperfusion with or without IPC. Black bars denote I/R groups, white bars reflect IPC + I/R groups. (n = 7 for all groups). Mean ± SEM, * P<0.05 vs. I/R in WT (ANOVA).

Next we examined whether the NLRP3 components affected the IPC-protective effects on mechanical function following I/R as observed in WT hearts (Fig. 1A and 1B). IPC significantly improved cardiac function following I/R in WT hearts, as demonstrated by diminished EDP and improved RPP recovery at end reperfusion. IPC was similarly effective in ASC^−/−^ hearts as in WT hearts, improving both EDP and RPP. Surprisingly, IPC was without effect on these parameters for the NLRP3^−/−^ hearts, indicating disrupted IPC signaling in these hearts. These data thus demonstrated that the NLRP3 component of the NLRP3 inflammasome is necessary for IPC protective effects on heart function, whereas the ASC component is not.

Subsequently, we examined whether the inflammasome components affected I/R and IPC effects on cardiac cell death. LDH release during reperfusion was used as index of cell death (Fig. 2). Thirty-five minutes of ischemia resulted in a large release of LDH during reperfusion for WT hearts. A similar release of LDH was observed for ASC^−/−^ and NLRP3^−/−^ hearts, indicating that deletion of these NLRP3 inflammasome components does not affect acute cell death induced by I/R. IPC significantly reduced LDH release in WT (−45%) and in ASC^−/−^ (−33%) hearts. However, IPC had no effect on LDH release in NLRP3^−/−^ hearts. Thus, although both ASC and NLRP3 protein do not affect I/R-induced cell death, the NLRP3 protein is necessary for IPC protective effects on cardiac cell death.

**Figure 6:**
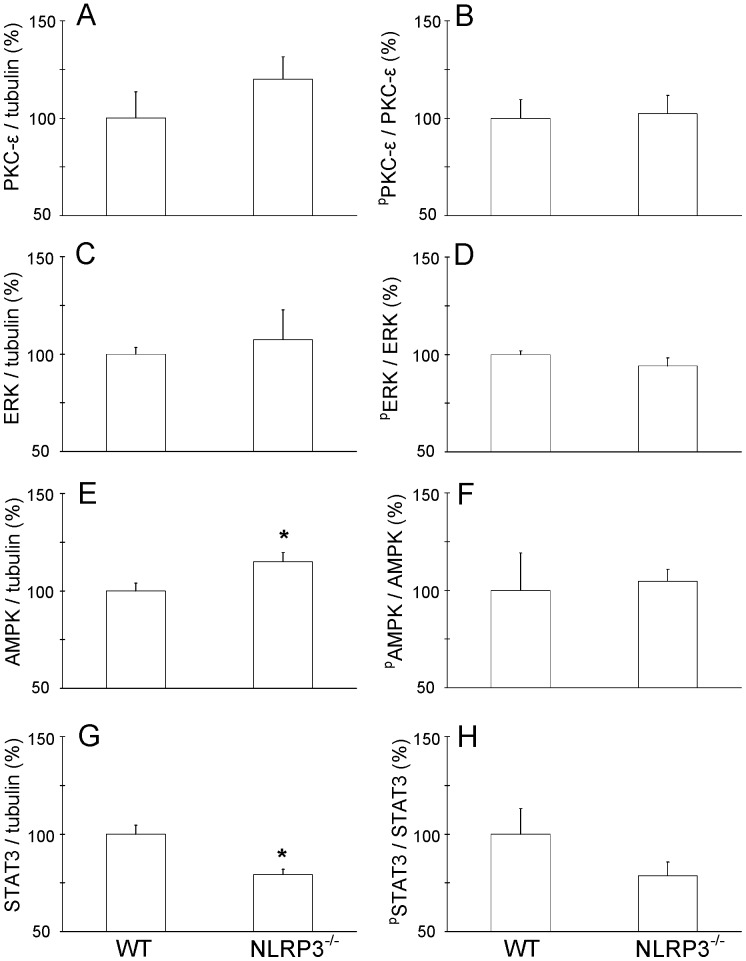
Decreased STAT3 and increased AMPK protein levels in NLRP3^−/−^ hearts. Effect of NLRP3 gene ablation on survival kinases and their phosphorylation status in preconditioned hearts analyzed at 5 min reperfusion following 35 min ischemia. PKC-ε (A), ERK (C), AMPK (E) and STAT3 (G), and their phosphorylation status (B, D, F, and H, respectively) are shown (n = 4 hearts per genotype). Mean ± SEM, *P<0.05 WT vs. NLRP3^−/−^.

Cytokines may be considered as inducers of IPC, in that small increases in cytokines can activate cardiac intrinsic mechanisms against I/R injury. To examine whether reduced cytokine levels were associated with the reduced IPC in NLRP3^−/−^ hearts, cytokine levels were determined in the reperfused WT, ASC^−/−^ and NLRP3^−/−^ - heart (Figure 3). Deletion of the ASC component had no effect on myocardial tissue levels of IL-6 (Fig. 3A), TNF-α (Fig. 3B) or IL-1β (Fig. 3C). Deletion of NLRP3 resulted in significantly decreased myocardial IL-6 levels and in a non-significant trend in decreased IL-1β levels. Ischemic preconditioning was without significant effects on any of the cytokine levels in the reperfused heart, despite a non-significant trend for decreased TNF-α levels in WT hearts. These data therefore show associations between reduced IPC and reduced cardiac IL-6 and IL-1β levels in NLRP3^−/−^ hearts.

To explore whether the observed directional decreased IL-1β and IL-6 levels in the NLRP3^−/−^ hearts may explain the IPC abrogation in NLRP3^−/−^ hearts, IPC was studied in WT hearts during inhibition of these pathways. To inhibit IL-1β signaling, the IL-1β membrane receptor IL-1R was inhibited through continuous perfusion with the IL-1RA Anakinra. However, IPC was still effective during IL-1R inhibition, neglecting an effect of IL-1R signaling in acute cardiac IPC. IPC + Anakinra decreased EDP (Fig. 4A), improved RPP recovery (Fig. 4B) and decreased LDH release during reperfusion (Fig. 4C) in WT hearts vs. I/R-only WT hearts.

To inhibit IL-6 signaling in WT hearts, IL-6 was neutralized through continuous perfusion with the anti-IL-6 antibody throughout the experiment. This treatment abrogated IPC: no improvement in EDP, RPP recovery or LDH release was observed (Fig. 4). The data demonstrate that IL-6 signaling is mandatory in IPC, and raise the possibility that diminished IL-6 signaling abrogates IPC in NLRP3^−/−^ hearts.

To further explore the possible cellular mechanisms of diminished IPC in NLRP3^−/−^ hearts, we determined HMGB1 mRNA levels in reperfused hearts. HMGB1 was reported as a preconditioning stimulus and was released in an inflammasome-dependent fashion [26]. However, no differences in HMBG1 mRNA levels in the reperfused hearts were observed between groups (Fig. 5), negating a role for HMGB1 in the attenuated IPC response in NLRP3^−/−^ hearts.

Finally, in a separate series of experiments we examined several survival kinases that have been associated with cardioprotection by IPC (Fig. 6). No differences in phosphorylation status of any of the survival kinases between preconditioned WT and NLRP3^−/−^ hearts at 5 min reperfusion were observed. There was a small increase in AMPK expression in NLRP3^−/−^ hearts versus WT hearts. The largest effect of NLRP3 gene knockout was however the diminished amount of STAT3 protein expression in preconditioned hearts, as compared to WT preconditioned hearts. The diminished STAT3 content, together with the reduced IL-6 content, suggest that the cardioprotective IL-6/STAT3 signaling pathway is reduced in the NLRP3^−/−^ hearts.

## Discussion

In this study we have demonstrated that the intracellular innate immunity NLRP3 component of the NLRP3 inflammasome is a required component of IPC in the heart. This role of NLRP3 in cardiac IPC is independent of the activation of the NLRP3 inflammasome, because ablation of the ASC-component of the NLRP3 inflammasome was without effects on IPC. In addition, our data suggest that the NLRP3-mediated cardioprotection is not transduced through extracellular IL-1β signaling, the cytokine that is most frequently associated with the NLRP3 inflammasome, but most likely through extracellular IL-6 signaling. Finally, the abolished IPC potential of NLRP3^−/−^ hearts was associated with a small increase in AMPK protein expression and a diminished expression of the survival kinase STAT3 in reperfused hearts.

The innate immune system consists of so-called pathogen recognition receptors that can be grouped in TLRs and NLRs [7]. Several studies have clearly demonstrated a role for TLRs in cardiac I/R injury, by showing that TLR deficiency resulted in diminished I/R injury [27]. However, a possible role for TLR in IPC has received much less attention. Only recently it has been shown that myocardial IPC requires TLR2 (but not TLR4) [28]. Our study shows for the first time that myocardial IPC also requires NLR. In contrast to TLR ablation, ASC or NLRP3 ablation did not result in diminished acute I/R-induced cell death. Cardiac functional recovery (RPP) was even partly decreased with ASC or NLRP3 ablation, suggesting divergent effects of the NLR constituents versus the TLR constituents of the innate immune pathway on cardiac I/R injury. However, our finding that NLRP3 is required for IPC cardioprotective effects is similar to the observation that TLR2 is needed for IPC [25]. Interestingly, only ablation of the NLRP3-component of NLRP3 inflammasome abrogated IPC, whereas ablation of the ASC-component of the NLRP3 inflammasome was without effect on IPC. It thus seems that it is a NLRP3 inflammasome-independent mechanism that is involved in cardiac IPC. Other recent reports [18], [19] support our observation that NLRP3 and ASC, both being crucial components of the so-called NLRP3 inflammasome protein complex, may affect cellular biology through inflammasome-independent mechanisms.

The significant decrease in IL-6 levels, which we have found in our NLRP3^−/−^ hearts, suggests IL-6 production to be, at least partly, regulated by NLRP3. We are unaware of other studies that have reported on IL-6 being dependent on NLRP3. As IL-18 was reported to induce IL-6 production, it is possible that diminished IL-18 in the NLRP3^−/−^ hearts mediated the lower IL-6 content [29]. Further studies will be necessary to delineate this association between NLRP3 and IL-6 cytokines at tissue levels. Most importantly, the similar myocardial IL-1β levels among NLRP3^−/−^ and WT hearts, together with the observation that inhibition of the IL-1β receptor with recombinant IL-1RA did not prevent IPC in WT hearts, makes it unlikely that the loss of IPC in the NLRP3^−/−^ hearts can be explained by differences in IL-1β signaling. In contrast, the decrease in myocardial IL-6 in NLRP3^−/−^ hearts together with the loss of IPC effects in WT hearts with a neutralizing antibody against IL-6, strongly suggest IL-6 to be the mediator of NLRP3^−/−^ -induced lost of IPC.

Although it is known that IL-6 is an integral part of the TNF-α/IL-1 cytokine signaling network [15], that TNF-α is involved in classical IPC [17], and that IL-6 plays an obligatory role in delayed preconditioning [16], we are unaware of studies that have demonstrated directly a role of IL-6 in early IPC of the heart. We now extend these discovered cardioprotective effects of IL-6 to early IPC, demonstrating that neutralizing IL-6 mitigates early IPC effects in WT hearts. Similar effects of IL-6 during IPC were recently observed in a model of hepatic ischemia-reperfusion [30]. Although in the current study IL-6 was not measured in the perfusate, other studies have shown that IPC can acutely increase tissue IL-6 mRNA and protein levels [16], [30]. Similar effects have been published for the cardioprotective cytokine TNF-α, which was acutely increased in plasma during the IPC-stimulus [31]. Interestingly, IL-6 has recently been indicated as a potential mediator of remote IPC [32], a cardioprotective intervention with demonstrated clinical benefits [33]–[35]. IL-6 binds to the transmembrane IL-6 receptor that is part of the larger gp130, which functions as a receptor subunit and signal transducer. Gp130 is involved in two major signaling pathways: the JAK/STAT3 and the SHP2-Erk-MAPK pathway [36]. Activation of the STAT3 pathway is now known to exert major cardioprotective effects against I/R injury, and is required in both early and delayed preconditioning schemes [37], [38]. Our analysis of survival kinases demonstrated that the abrogated IPC-effects in the NLRP3^−/−^ hearts was associated with reduced STAT3 protein expression, at least suggesting that reduced signaling through this IL-6/STAT3 pathway is a likely candidate to explain the reduced IPC-protective effects in NLRP3^−/−^ hearts. Interestingly, recent studies have demonstrated that IL-6 is a potent activator of STAT3, providing a direct link between this pro-inflammatory cytokine IL-6 and STAT3 [39], [40]. The observed reduced amount of STAT3 in NLRP3^−/−^ hearts may also have its implications for other studies using these mice, provided that STAT3 is also reduced in other organs/tissues of NLRP3^−/−^ animal.

A potential limitation of the use of the NLRP3 and ASC germ line knockout strains, is that from these presented data we cannot exclude the possibility of partial functional redundancy of Nlrp3 or ASC with other inflamasome componens like e.g. NLRP4 and others. These data could also indicate that Nlrp3 may have some functional redundancy. Future studies of double and triple knockout animals may be of value in addressing these issues. A further limitation of our present study is its ex vivo nature, excluding the possibility of studying effects of NLRP3/ASC ablation on acute cardiac I/R and IPC through blood borne components such as e.g. leucocytes. However, such an approach will also allow dissection of organ intrinsic regulatory mechanisms concerning I/R injury relative to effects mediated through interaction with blood-born components derived from e.g. bone-marrow, ultimately resulting in a full comprehension of innate immunity effects on I/R injury. Such differences may explain why NLRP3^−/−^ deletion was beneficial in an in vivo model of kidney I/R injury [12], probably as a result of decreased leucocyte recruitment in the injured organ of the NLRP3^−/−^ animal. A recent study in the in vivo model of myocardial I/R injury also demonstrated that inflammasome activation contributed to in vivo I/R cardiac injury [41]. Ischemic preconditioning was however not examined in that study. Further studies in an in vivo model will be needed to answer these questions for the heart. The current study has clearly demonstrated that for the heart only, ablation of the innate immunity protein NLRP3 prevents cardiac IPC signaling, probably through diminished cardiac IL-6/STAT3 signaling.
